# Quality of sleep in women with menopause and its related factors

**DOI:** 10.5935/1984-0063.20220021

**Published:** 2022

**Authors:** Fatemeh Ahmady, Maryam Niknami, Zahra Bostani khalesi

**Affiliations:** School of Nursing and Midwifery, Guilan University of Medical Sciences, Rasht, Iran.

**Keywords:** Menopause, Women, Sleep, Postmenopause, Dyssomnias

## Abstract

**Background:**

Menopausal period is one of the most critical stages of a womans life. Complications of the menopausal period including sleep disorders can affect the physical and mental state of women. As sleep disorder has a determinant role in the quality of life, this study was conducted to evaluate postmenopausal womens quality of sleep and its related factors.

**Material and Methods:**

This cross-sectional-analytical study was conducted on 323 postmenopausal women based on convenience and consecutive sampling. The data-gathering tool consisted of two parts; sociodemographic characteristics and the Pittsburgh Sleep Quality Index (PSQI). Data analysis was performed using descriptive and inferential statistical tests at a significance level of p<0.05.

**Results:**

Sleep disorder was determined in 49.9% of participants. The mean PSQI score was 5.32 ± 3.881. There was a significant correlation between PSQI and age (ß = 0.29, p < 0.001) indicating that sleep disorder increased with an increase in age. There was a significant correlation between body mass index (ß = 0.599, p < 0.001) and undesired sleep quality.

**Conclusions:**

Regarding the presence of sleep disorder in almost half of the study participants, and the relationship between sleep quality and body mass index and age, it is recommended that decision and policymakers design educational consultation interventions to improve the quality and quantity of sleep in menopause women.

## INTRODUCTION

Menopause is an important phenomenon in women’s life that is associated with loss of fertility and transition to a new status in life^1^. Natural menopause is defined as a lack of menstruation for one year^1,2^. The mean age of menopause is 51 years old in the world^3^ and 49.26 years old in Iran. The mean menopause age in Iran is lower than in developed countries^1,2^.

In recent decades, a large number of women enter the menopausal period daily due to the developments in medical science and an increase in life expectancy^4^. It is predicted that the population of menopausal women will reach 1.2 billion in 2030 with an annual 47 million new menopause cases. Currently, the population of women over 45 years old in Iran is approximately 155000^5^.

Reduced body hormones in the menopausal period result in various changes including fushing, nocturnal perspiration, palpitation, headache, confusion, fatigue, and irritation. One of the most common problems in this period is a sleep disorder^2^. Change in sleep pattern is associated with hormonal status including estrogen. Estrogen receptors are present in the central nervous system^6^. Serotonin level, which is also an important factor in the sleep process, is reduced along with reduced estrogen in the post-menopausal period. Reduced serotonin can also lead to T sleep disorders^7^. Furthermore, fushing due to a sudden drop in estrogen level may result in turmoil in women’s workplace, suspension of daily activity, and change in sleep pattern of women^5^. Prevalence of sleep disorder in menopausal women is reported to range from 14 to 65 percent^8^. Sleep disorders in postmenopausal women can be chronic or temporary and may present from minimal disturbance to severe and debilitating symptoms^9^. Furthermore, sleep disorder mainly presents itself as difficulty in falling asleep, frequent waking at night and disorder in sleeping after waking up, and frequent awakening^10^.

Sleep is a complicated behavior that is vital for healthy body function. Sleep quality is a complex phenomenon that is difficult to define^11^. Definitions of sleep quality are subjective and therefore, cannot be assessed in the laboratory. Sleep quality is a subjective index that is related to the quality of sleep experience, including sleep satisfaction, and an individual is feeling after awakening^4^. Based on the theory by the international sleep foundation, adequate sleep is around 7 to 8 hours that is crucial for cognitive function in adults. Sleep is the time of rest for the brain and body, during which the level of consciousness is reduced^8,10^.

Sleep phenomenon reduces stress and refreshes the mental, psychological and physical condition of an individual^12^. Although the mechanism of the benefits of sleep for the body is not yet fully understood, sleep has always been regarded as an essential need of humans^13^. Quality and quantity of sleep can affect learning, memory, and various cognitive abilities, especially activities that are related to memorizing new information and learning new skills in education environments^9^. Undesirable sleep quality may cause daytime sleepiness, mood alteration, and increased risk for unhealthy behaviors, including drug abuse^14^. The sleep disorder can increase mental and psychological disorders, cognitive performance, learning disorder, fatigue, problem in performing the job and educational responsibilities, and physical problems as well as quality^15^.

The main objective of this paper is to find out the postmenopausal women’s quality of sleep and its related factors. Even though many researchers were worked on sleep disorders in postmenopausal women, very few researchers have reported the related factors of quality of sleep. Identify the related factors that are the main criterion for designing health interventions can provide a normative framework for efficient intervention. These data are very useful in the design of Interventions for improving sleep quality in menopausal women. In the present work, the prevalence of menopausal women’s sleep disorders and related factors in Guilan is studied exclusively.

This study was conducted to evaluate postmenopausal women’s quality of sleep and its related factors.

## MATERIAL AND METHODS

This study was a cross-sectional-analytical correlation study on 323 postmenopausal women older than 45 years old who met the inclusion criteria. The inclusion criteria were willingness to participate in the study, ability to read and write, being menopause for at least one year, lack of sleep disorders before menopause (based on self-report), not receiving estrogen and progesterone hormones, not receiving psychological medications (based on self-report), and negative history for severe psychological stress, including experiencing accidents or loss of first degree relatives during the past 3 months. The exclusion criteria were incomplete questionnaires. Sampling was based on convenience consecutive sampling.

Ethical clearance was obtained from the Deputy of Health of Guilan University of Medical Sciences (Code: IR.GUMS.REC.1399.076, Approval Date: 2020-05-27). Then, the researcher introduce, explaining the study objectives and how to respond to the questionnaire, ensuring the confidentiality of information, and Informed consent was obtained from all participants prior to data collection. Consent forms assured anonymity for all participants with the following caveats: exit survey participants were told that their name and contact number would be requested in a separate form if they were referred.

The data-gathering tool consisted of two parts. Sociodemographic characteristics form included information regarding age, age at menopause, level of education, spousal age, level of education, and occupation; economic status, place of living, household number, number of children, marital status, gravida, number of post-menopausal years. The second part of the data-gathering tool was PSQI^16^. The PSQI is used to detect sleep disorders during the past month. PSQI consists of seven subscales, including subjective sleep quality, sleep latency, sleep duration, habitual sleep efficacy, sleep disturbance, use of sleeping medication, and daytime dysfunction. PSQI items are scored based on a four-point Likert scale ranging from zero to three. The total score is calculated by summing up the scores of the subscales. The PSQI score may range from zero to 21. Sleep disorder is defined as PSQI scores equal to or higher than five. This indicates that scores 0 to 4 refect lack of sleep disorder, scores 5 to 10 refect mild sleep disorder, scores 11 to 16 refect moderate sleep disorder and scores 17 to 21 refect severe sleep disorder. The reliability of the test according to Cronbach’s alpha was 0.81. In addition, the internal consistency of the PSQI was 0.81 and the scales correlation score ranged from 0.48 to 0.71^17^.

### Statistical analysis

Continuous variables were presented using mean and standard deviation, while categorical variables were presented using frequency and percentage. Data analysis was performed using parametric tests and Non*-*parametric tests (Student’s t-test, Mann*–*Whitney U test, one*-*way analysis of variance (ANOVA), Kruskal-Wallis H test) based on normality of data and multivariate linear regression. The normality of data was assessed using the Kolmogorov-Smirnov test in order to choose an independent t-test and one-way analysis of variance. The Fisher’s exact test was used to compare PSQI scores between personal factors in participants. Multivariable analysis was performed controlling for confounders using multivariate logistic regression models (odds ratio). Data were analyzed using the statistical package for social sciences (SPSS for Windows, Version 16.0. Chicago, SPSS Inc). The level of statistical significance was considered as 0.05.

## RESULTS

The mean age of the participants was 57 ±2.11 years. Experience of at least three pregnancies was reported in 36.8% of the participants. The majority of postmenopausal women (51.7%) reported that their menopausal age was between 51 to 55 years old. Daily perspiration and fushing were reported in 49.8% of the participants mostly during the daytime. Regarding the menopausal initiation time, the majority of participants (61%) reported their menopausal duration was at least 4 years. In terms of alcohol abuse, 98.1% of the participants reported that they did not drink alcoholic drinks. Among the chronic diseases, the skeletal disease was present in 13.9% of the participants. Cardiovascular disease, diabetes, and other chronic diseases were reported by 9.6%, 24.1%, and 11.8% of the participants, respectively (Table 1).

**Table 1:** Frequency distribution of the study participants based on personal, social, and fertility indices (N=323).

Variable	Category	Frequency	Percentage
Age group	45-50 years old	18	5.6
	51-55 years old	111	34.4
	56-60 years old	143	44.6
	61 years and older	51	15.8
Education level	Below high school	97	30
	High school graduate	139	43
	University degree	87	26.9
Marital status	Single	38	11.8
	Married	201	62.2
	Divorced	8	2.5
	Widowed	76	23.5
Duration of menopause	1-2 years	35	10.8
	2-4 years	91	28.2
	4 or more years	197	61

Based on one-sample t-test, 51.1% of the menopausal women did not have sleep disorder, 38.1% had mild sleep disorder, 9% had moderate sleep disorder, and 1.9% had severe sleep disorder. Based on the distribution table, the chi-square value was 212.36 with the degree of freedom of 3 and level of significance <0.001, which was considered significant based on 95% confidence interval and 5% type one error. In other words, frequency distribution and concentration were high in some levels of sleep disorder spectrum. Majority of the participants were categorized in no sleep disorder and mild sleep disorder categories.

There was a significant correlation between sleep quality and age, caffeine use and nocturnal perspiration, cardiovascular disease, physical activity, and exercise.

Based on the “Fisher’s exact test”, there was a significant correlation between sleep disorder and body mass index. In other words, higher body mass index was correlated with more sleep disorder.

Based on the regression model, body mass index and age had a good power to predict sleep disorder. Table 4 shows that body mass index and age, respectively had the highest infuence on sleep disorder and caffeine use, nocturnal perspiration, and finally exercise had the least effect on sleep disorders. According to the results of the application of regression method, it is observed that the significance level of the hypothesis of the ineffectiveness of body mass index and age of each separately on sleep disorders is 0.00 and less than 1% error, in addition to significant confirmation The regression model of this table shows that the greatest effect on sleep disorder was by body mass index and age, then caffeine consumption, night sweats and finally exercise had the least effect. The positive sign of the standard coefficient indicates a direct relationship between the variables of body mass index and age with the criterion variable (sleep disorder), in other words, if the body mass index and age increase, sleep disorders become more.

**Table 4:** Factors related to sleep disorder among participants.

Variable		F	Sig	Unstandardized coefficient		Standardized coefficient		
				β	Standard error	β	t	Sig.
Constant				−27.26	1.63	-	−16.71	<0.001
Body mass index				0.59	0.052	0.599	11.5	<0.001
Age				0.26	0.04	0.29	5.77	<0.001
Cardiovascular disease	Z1			0.29	0.36	0.023	0.82	0.408
Dummy variable Caffeine use	Z2			−1.61	0.56	−0.205	−2.85	0.005
	Z3	222.03	<0.001	−1.21	0.73	−0.159	−1.64	0.101
Dummy variable Nocturnal perspiration	Z4			1.94	0.63	0.193	3.04	0.003
	Z5			1.07	0.57	0.11	1.88	0.061
	Z6			1.72	0.70	0.226	2.43	0.016
Dummy variable Exercise	Z7			0.61	0.24	0.08	2.48	0.013
	Z8			0.002	0.288	<0.001	0.006	0.995

## DISCUSSION

The current study was performed to determine sleep quality and define its related factors among postmenopausal women. The findings of the current study showed that half of the postmenopausal women who participated in the study had a sleep disorder. On the other hand, age and body mass index had the highest predictive power for sleep quality in menopausal women. In the study by Lampio et al., more than 70% of postmenopausal women suffered from insomnia^18^. This controversy can be justified by the existence of confounding variables including lifestyle, consumption of some medications, and herbal medicine, as traditional medicine, in participants in different countries. These confounders can affect sleep quality. In a cross-sectional study by Azhari et al. on 400 menopausal women who referred to the Gynecology clinic of educational centers in Mashhad, Iran, 73% of the participants had an undesirable sleep disorder^19^. Sociodemographic characteristics including the level of education, marital status, occupation, economic status, number of children, and gravida did not have a significant correlation with sleep disorder among menopausal women. However, it seems that some of the studied variables including economic status and level of education can affect sleep quality. The findings of a study showed that individuals who had acceptable economic status and thus utilized sports facilities and had higher physical activity had a better sleep quality compared to those with lower economic status due to the positive effect of physical activity on sleep quality. Furthermore, regarding the correlation between age and sleep quality in menopausal women, a study showed that age was not only correlated with the timing of awakenings after falling asleep and the minimum body temperature, but also the irregularity and shortness of sleep duration was correlated with the pace of increase in body temperature. It was determined that melatonin possesses its sleep inductive effects through changes in central body temperature^20^. Furthermore, women encounter various changes including fushing, nocturnal perspiration, palpitation, headache, confusion, fatigue, and irritability at menopause due to reduced body hormones. These changes result in frequent awakening and result in undesirable sleep quality^21^. The findings of the current study showed that variables including age and body mass index had the highest predictive power for sleep disorder among menopausal women^22,23^. Some studies on the evaluation of the relationship between age and sleep quality have demonstrated that sleep quality reduced with an increase in age^24,25^. This controversy could be rationalized by the fact that these correlations were related to developmental changes that happen during the life of adults, which includes increased invulnerability of the sleep-wake rhythm regulating system and can therefore affect sleep quality. However, it is not clear when age-related changes in sleep quality can be considered as sleep disorders^26^. On the other hand, other findings indicated that body mass index had the highest predictive power for THE sleep disorder. This finding was in line with the findings of the study by Fanfulla et al., which indicated a direct correlation between body mass index and sleep disorder. This finding indicated that sleep disorder increases with an increase in body mass index^27^. Furthermore, it is obvious that fat mass surrounding the neck increases with an increase in body mass index, thus changes the upper respiratory airway, and makes breathing difficult during sleep. Similarly, de Melo et al. showed that high consumption of foods during the day decreases sleep quality in patients^28^.

## CONCLUSIONS

The findings of the current study showed that half of the postmenopausal women who participated in the study had a sleep disorder. On the other hand, variables including age and body mass index had the highest predictive power for sleep quality among menopause women. In other words, increased age and body mass index were correlated with the increased sleep disorder.

## Figures and Tables

**Table 2:** Sleep disorder severity and sleep quality among participants.

Sleep disorder severity	Observed frequency	Observed percentage		Predicted percentage	
no sleep disorder (0-4)	165	51.1		80.8	
mild sleep disorder (5-10)	123	38.1		80.8	
moderate sleep disorder (11-16)	29	9		80.8	
severe sleep disorder (17-21)	6	1.9		80.8	
χ^2^		212.36			
Degree of freedom		3			
p-value		<0.001			
T test	Mean	Standard deviation	t	Degree of freedom	p-value
Sleep quality	5.32	3.8810	− 26.77	322	<0.001

**Table 3:** Sleep disorder based on sociodemographic characteristics.

Variables		No sleep disorder		Mild sleep disorder		Moderate sleep disorder		Severe sleep disorder		Total		*P* value
Age group	45-50 years	18	5.57	0	0	0	0	0	0	18	5.57	0.902
	51-55 years	111	34.3	0	0	0	0	0	0	111	34.3	
	56-60 years	34	10.5	108	33.4	0	0	1	0.31	143	44.2	
	61 years and older	2	0.62	15	4.64	29	8.98	5	1.55	51	15.7	
Marital status	Single	26	8.05	10	3.1	2	0.62	0	0	38	11.8	0.12
	Married	104	32.20	76	23.53	16	4.95	5	1.55	201	62.2	
	Divorced	3	0.93	4	1.24	1	0.31	0	0	8	2.5	
	Widowed	32	9.1	33	10.22	10	3.1	1	0.31	76	23.5	
Occupation	Medical fields	19	5.8	10	3.1	3	0.93	0	0	32	9.91	0.78
	Non-medical fields	146	45.2	113	34.37	26	8.05	6	1.86	291	90.0	
Economic status	Good	38	11.76	38	11.76	10	3.1	1	0.31	87	26.9	0.09
	Moderate	82	25.3	51	15.7	13	4.02	4	1.24	150	46.4	
	Poor	45	13.9	34	10.53	6	1.86	1	0.31	86	26.6	
Number of children	No child	18	5.57	9	2.79	1	0.31	0	0	28	8.68	0.091
	1 child	14	4.33	20	6.19	8	2.48	1	0.31	43	13.3	
	2 children	48	14.8	40	12.38	11	3.41	3	0.93	102	31.5	
	3 and more children	85	26.32	54	16.72	9	2.79	2	0.62	150	46.4	
Gravida	Never	19	5.88	10	3.1	2	0.62	0	0	31	9.6	0.41
	1	29	8.98	34	10.5	10	3.1	2	0.62	75	23.2	
	2	52	16.1	34	10.5	10	3.1	2	0.62	98	30.3	
	3 or more	65	20.1	45	13.93	7	2.17	2	0.62	119	36.8	

Sleep disorder based on sociodemographic characteristics were assessed using Fisher’s exact tests.
